# Molecular dynamics simulation analysis of Focal Adhesive Kinase (FAK) docked with solanesol as an anti-cancer agent

**DOI:** 10.6026/97320630013274

**Published:** 2017-09-30

**Authors:** Betty Daneial, Jacob Paul Vazhappilly Joseph, Guruprasad Ramakrishna

**Affiliations:** 1 Botany Department, St. Joseph's College, Bangalore 560027, Karnataka, India; 2 Durga Femto Technologies and Research, Bangalore 560018, Karnataka, India;

**Keywords:** FAK, solanesol, angiogenesis, blind docking, contact scoring, GROMACS

## Abstract

Focal adhesion kinase (FAK) plays a primary role in regulating the activity of many signaling molecules. Increased FAK expression has been associated in a series of cellular processes like cell migration and survival. FAK inhibition by an anti cancer agent is critical. Therefore, it is of interest to identify, modify, design, improve and develop molecules to inhibit FAK. Solanesol is known to have inhibitory activity towards FAK. However, the molecular principles of its binding with FAK is unknown. Solanesol is a highly flexible ligand (25 rotatable bonds). Hence, ligand-protein docking was completed using AutoDock with a modified contact based scoring function. The FAK-solanesol complex model was further energy minimized and simulated in GROMOS96 (53a6) force field followed by post simulation analysis such as Root mean square deviation (RMSD), root mean square fluctuations (RMSF) and solvent accessible surface area (SASA) calculations to explain solanesol-FAK binding.

## Background

Cancer has affected more than 15 million people across the globe and it is expected to affect 25 million people in the next 20 years [1]. Cancer is regarded as the fastest progressing noncommunicable disease with frequent appearance of numerous new forms every year, which are often resistant [2]. Even though massive hard work have been put by researchers, healthcare professionals, etc. in discovering, developing, and utilizing chemotherapeutic approaches for managing neoplasm, but still it continues to be a serious issue across the world at present. The identification of several unexplored classes of cell-cycle regulators and apoptotic stimuli were the emerging strategy in anti-cancer drug discovery. At present, a number of inhibitors have been discovered which modulates cellular resistance, hormonal milieu, angiogenesis, cellular migration, cell proliferation, and DNA targets [3]. The inhibition of angiogenesis (preventing new blood vessels formation) has been representing as an impressive strategic approach in managing cancer and which in turn prevention of metastasis.

Angiogenesis is the process in which new blood vessels originate from the existing blood vessel system [4]. Innumerable proangiogenic factors such as focal adhesion kinase (FAK), TGF-b, vascular endothelial growth factor (VEGF), angiogenin, fibroblast growth factor (FGF), and many other factors, also known as "angiogenic switch" promote constitution of new vessels [5]. In the case of tumors, these factors get over-expressed, that eventually enhances tumor growth and metastasis [6]. From last few years, various researches have revealed successful restriction of tumor proliferation by preventing accessibility of nutrient supply to the highly metabolizing cell like cancer [7]. The inhibition of these pro-angiogenic factors like FAK will lead to a halt in tumor progression.

Focal adhesion kinase (FAK) is a cytoplasmic tyrosine kinase (PTK2) that plays a primary role in growth factor mediated signaling and mediates significant role in cell migration, proliferation, and angiogenesis [8]. Several vascular growth promoting factors like insulin-like growth factor (IGF)-I, vascular endothelial growth factor (VEGF), and basic fibroblast growth factor (bFGF) activates FAK [9]. FAK have also been identified recently to be the prime factor in retinal angiogenesis [10]. Any mutation or alterations in the expression of FAK results in the generation of tumors promote metastasis and endorse vascular growth [11]. FAK has been associated with several forms of cancers like breast, colon, ovarian, prostate, head and neck, thyroid, oral, stomach, cervical, liver, sarcoma, melanoma, and glioblastoma [12]. It has been reported that blockade of FAK often results in reduction of metastasis and mobility in case of breast cancer [13].

Natural products have a huge reputation as promising anticancer agents and in the modern era, naturally derived products are frequently accepted in therapy among the masses [14]. A lot of dietary isoprenoid-based compounds have come into limelight owing to their chemopreventive and antiproliferative properties [15]. Some less known isoprenoid derivatives are now finding applications in mainstay chemotherapy [16]. Solanesol is believed to be among one of the most eligible candidates to demonstrate anti-cancer activity. Commercially, solanesol gets extracted from the tobacco plant, the richest source. As evidenced and rejuvenating the fact from several famous English literature that "Mother Nature inculcates all miraculous remedies in it", our search for an unexplored class ended in tobacco. In one-half, the American Cancer Society (ACS) sticks to the fact that tobacco is the leading cause of cancer. On the other hand, solanesol, the chemopreventive, anti-angiogenic and anti-tumor principle is obtained from the same plant, tobacco, which is quite mysterious and highlights the immense beauty of nature.

Solanesol is a non-cyclic polyisoprenoid alcohol (composed of nine isoprenoid unit) present mainly in solanaceous crops like tobacco, tomato, potato, eggplant, and pepper plants [17]. Solanesol appears as a waxy white solid at room temperature, it is optically inactive, non-polar in nature and exhibit solubility in like-wise non-polar solvents soluble [18]. Solanesol finds application as the intermediate for the synthesis of ubiquinone drugs, such as coenzyme Q10, vitamin K2 and vitamin E [19]. It known to possess activities like antimicrobial [20], antioxidant [21], antiviral [22], anticancer [23], anti-inflammatory, and antiulcer [24] activities, and its derivatives also have anti-oxidant and anti-tumor activities, in addition to other bioactivities. Derivatives of Solanesol are known anti-cancer agents [22].

The present research involves establishing of solanesol as a focal adhesion kinase (FAK) inhibitor by applications of computational in silico methods. Though, solanesol used as a bioactive agent in industries for decades, due to its highly flexible nature, there is no successful in silico protein binding and simulation data available online till date. We also proposed a method of binding of the highly flexible compound to protein targets using an enhanced contact based scoring method. This method scores the residues rather than the conformations. The higher scored residues were then used for more "focused" docking on those residue regions.

## Methodology

### Protein selection and preparation

The crystallographic co-ordinates for Focal adhesion kinase (PDB ID: 4Q9S) [25] were retrieved from the Protein Data Bank (PDB). Prior to docking, protein structures were prepared by removing water molecules using UCSF Chimera software [26]. Following which, bond orders were assigned, and hydrogen atoms were added to the crystal structures.

### Ligand preparation

Solanesol exist in both cis and trans states, for this experiment we considered only trans is found in natural sources [27]. The structure of Solanesol was obtained from PubChem compound (CID 5477212). Gaussian 09 program was used to obtain the optimum geometry of the structures using the density function theory at the B3LYP/6-31G (d,p) level [28].

### Molecular docking

All the molecular docking studies of Solanesol to FAK were performed using Autodock 4.2 [29]. Autodock uses a semiempirical free energy force field to evaluate binding conformations of ligand while docking. The AutoDockTools was used for preparing protein and ligand parameters files. For ligands; the hydrogens, compute Gasteiger charges, and nonpolar H atom were added which ensuring the total charge corresponds to the tautomeric state followed; and, at last torsion tree root and rotatable bonds were chosen. For macromolecules; hydrogens, compute Gasteiger charges, and merge non-polar H were added which was followed by Stouten atomic salvation parameters assignment; and at last flexible residues PDBQT in addition to the rigid PDBQT file were created. A RMSD value inferior or close to 2Å was considered as a successful docking [30].

### Binding site analysis

Solanesol is a 45-carbon chain with 26 rotatable bonds. As it is extremely flexible it is hard to determine the bind mode of it with the protein. The commonly used protocol for determination of binding pocket is "Blind docking", which was initially developed for to determining peptide docking with protein [31]. In this method the constrained ligand (or peptide) is docked with the whole protein surface. The place where it forms a cluster with higher energy determines the binding site. Then these sites were used for "refined docking" where the lowest binding modes for each of these places (in case if there are more than one) where determined by molecular mechanics and molecular dynamics studies.

For calculating the possible area of interaction or binding site of a highly flexible ligand we enhanced blind docking using Ligand Contact Based Scoring function for Residues (LCBSR). This is an atomic contact/clash based scoring method, in which the residues are scored based on the higher favorable interactions and probability of formation of a hydrogen bond. As it doesn't depend on the clustering or solely on binding energy, it statistically enhances the probability of finding the possible binding site.

It can be represented as,

LBCSR = log(NCo - NCl) - log(NH/CCl) (1)

Where, ligand contact based scoring of residue (LCBSR) is calculated using "Number of Contacts" (NCo) which is the number of occurrences where atoms of residue (r) are in contact with any atom of the ligand in all the conformations. "Number of Clashes" (NCl) is the number of occurrences when atoms of residue (r) are in clashes or unfavorably overlapped with any atom of the ligand in all the conformations. "Number of Hydrogen Bonds" (NH) is the number of occurrences when atoms of residue (r) are forming a hydrogen bond with any atom of the ligand in all the conformations. "Number of Clashes" (CCl) is the number of conformers where residue (r) is in an unfavorable overlap with any atom of the ligand.

The "Contact" here is defined as the instance when the difference between the distance of two atoms and the sum of their van der Waal radii is 0.4 A or more or, in other words, the distance is greater than the sum of van der Waal radii (Eq. 1) of two atoms. Whereas "Clash" is defined as the condition where the van der Waal radii of two atoms unfavourably overlaps each other and the distance is lower than the sum of radii (Eq. 2) of the two atoms.

This can be represented as:

∑rVDW(i,j) - Dij ≥ -0.4 (2)

∑rVDW(i,j) - Dij ≥ 1.0 (3)

Where, ∑rVDW(i,j) is the sum of van der Waal radii of interacting atoms of ligand (i) and residue (j) and Dij is the distance between interacting atoms of ligand (i) and residue (j). A Higher value of LCBSR of any residue implies the residue may be a part of the binding pocket for the ligand and, if it is capable, it may also form a hydrogen bond with the ligand. Lesser score implies a lower interaction or high chances of unfavorable clashes or low chances of forming a hydrogen bond.

### Blind docking

Solanesol has 25 rotatable bonds, which makes very difficult to dock directly to the protein structure by conventional method. Although, there are various methods for prediction of binding site in FAK, the flexible nature of solanesol as well as the size of the compound makes it difficult to fit the active site. For the purpose of predicting the actual binding site, blind docking method can be used. Blind docking method was introduced for the purpose of docking peptide molecules to the protein molecule but is a tested method for binding of small molecules when binding site is unknown [32]. Solanesol was docked against FAK structure (PDB ID: 4Q9S) using Autodock4.2 three times (as mentioned in materials & methods). For all the three times, a grid of size 126 x 126 x 126 with 0.375A spacing was created with protein center (Centre coordinates = 9.7, 0.16, 15.1) as the grid center, as per the normal "blind docking" protocol. Dielectric constant value was kept default. The method of blind docking comprise of locking of ligand molecule's all the torsions, for the purpose of reducing calculation time, and then docking on the complete protein surface. Then the best cavity or binding pocket is selected, based on the clustering of highest binding affinity conformations. In this experiment, Solanesol was docked a total of 3 times (Table 1) with "Blind Docking" protocol. For getting an unbiased result, all the rotatable bonds were kept unconstrained for all the experiments. The protein was covered using a 126 x 126 x 126 grid box with protein centre as grid centre. For experiment ligand-starting position was changed. The docking resultant file from Autodock was then converted into multiple PDB files using Autodock scripts. All the contacts and clashes, as well as hydrogen bonds between the conformation and protein, were calculated using UCSF Chimera tool. Considering all the conformers may lead to false positives, thus conformers were separated in three criteria: (1) binding energy less than -2.0 kcal/mol; (2) binding energy less than -3.0 kcal/mol; and binding energy less than -4.0 kcal/mol. Provided - 4.0 is roughly half the average of the binding energy (Average B.E = -7.10 kcal/mol) of all the three experiments, ensuring only conformations with low B.E were considered. The resultant values from each of these three were added to corresponding residues. This way the residues interacting more with the conformations of better binding energy will have a better score. For getting a statistically significant result, all the scores of the residues from all the three experiments were added to get the final score of each residue. Only those residues, which appeared in more than 2 experiments, were considered.

### Refined docking

Binding site for Solanesol was considered using residues with a higher LCBSR score. This binding site was then used for refined docking using Autodock. The experiment was done twice with (1) relaxed parameters, GA maximum energy evaluations 2.5 x 106, for 200 GA runs (2) exhaustive parameters, GA maximum energy evaluations 3.5 x 107, for 200 GA runs.

### Knowledge-based rescoring

All conformations were rescored using DSX, Drug Score eXtended [33], knowledge-based rescoring based on the DrugScore formalism, to estimate the affinity of conformation for FAK. The best conformations were selected based on the rescored values. The best conformation bound complex of FAK was further used for molecular dynamics simulation studies.

### Molecular dynamics

Molecular dynamics simulations for FAK protein as well as Solanesol bound FAK were performed using the GROMACS (Groningen Machine for Chemical Simulations) 4.6 [34] software with GROMOS96 (53a6) force field. PRODRG [35] server was used to generate topology files for Solanesol. Charges were kept full and no energy minimization was done using PRODRG. The complex was solvated in a dodecahedron box with SPC model water model molecules and periodic boundary conditions were used. One negatively charged chlorine ion (Cl-) was added to the system for maintaining the system's neutrality. The Lincs and Shake algorithm [36] were used for constraining bond length and fixing all bonds containing hydrogen atoms respectively.

For electrostatic calculations, Particle Mesh Ewald (PME) [37] method was used, with a coulomb cutoff of 1.2 nm, Fourier spacing of 0.16 nm and an interpolation of order 4. Energy minimization of the system was carried out using steepest descent algorithm with a tolerance value of 1000 kJ mol-1nm-1. After energy minimization, NVT and NPT equilibrations were done on the system until it reached the room temperature and water density. Production MD was performed for 20 ns time duration for both the simulations.

### Molecular dynamics trajectories analysis

The root mean square deviation (RMSD) and root mean square fluctuations (RMSF) of FAK backbone were calculated using "g_rms" and "g_rmsf" utility commands, respectively. A spherical probe of radius 1.4 Å across the protein surface was used for calculating solvent-accessible surface area (SASA) by "g_sasa" tool of Gromacs. Hydrogen bonds between Solanesol and FAK were calculated using "g_hbond" tool with proton donor and acceptor distance ≤ 3.5 Å and the angle between acceptor-donor-hydrogen ≤ 30.0 degrees.

### Binding free energy calculations

The molecular mechanics Poisson-Boltzmann surface area (MMPBSA) [38] approach was to estimate the binding free energy of protein-ligand interaction. For this purpose, "g_mmpbsa" [39] tool was used. The tool calculates the molecular mechanics potential energy and the free energy of solvation and excludes the entropy calculations. MM-PBSA calculations were performed using 1000 snapshots taken from last 5 ns of trajectories of the complex system. The MM-PBSA based binding affinity (ΔΔG) was calculated using the g_mmpbsa provided script.

ΔΔGBE=ΔGComplex- (ΔGReceptor + ΔGLigand) (4)

ΔG = ΔEMM + ΔGSol - TΔS (5)

ΔEMM = ΔEint + ΔEele+ ΔEvdw (6)

ΔGSol = ΔGPB + ΔGSA (7)

ΔEMM, ΔGSol and TΔS represent the molecular mechanics component in the gas phase, stabilization energy due to salvation, and a vibrational entropy term, respectively. ΔEMM is the summation of ΔEint, ΔEcol, and ΔEvdw, which are the internal, coulomb, and van der Waals interaction terms, respectively. ΔGsol is the salvation energy and it is divided into an electrostatic salvation free energy (GPB) and a non-polar salvation free energy (GSA).

## Result and Discussion

### Docking analysis: Blinding docking and LBCSR Score

Solanesol shows very small cluster with insignificant binding affinity towards FAK when docked "blindly" (Table 1). Though, the structure shows quite high binding affinity towards the kinase but the conformations with higher binding energy fails to form any significant cluster.

From all the generated conformations of Solanesol, which were having binding energy lower than -4.0 kcal/mol, all the favorable and unfavorable overlaps of the atoms were calculated using "FindClash" tool of UCSF Chimera. Chimera tool, "FindHbond" was also used to find the hydrogen bonds between the ligand and residue atoms. The default values were kept for all the calculation in Chimera.

An in-house python script was used to calculate the number of "Clashes", "Contacts" and "Hbonds" between all the conformations and residues as well as individual scores. The scores from all the three experiments were added to give a final score for each residue (Table 2).

**Figure 2:**
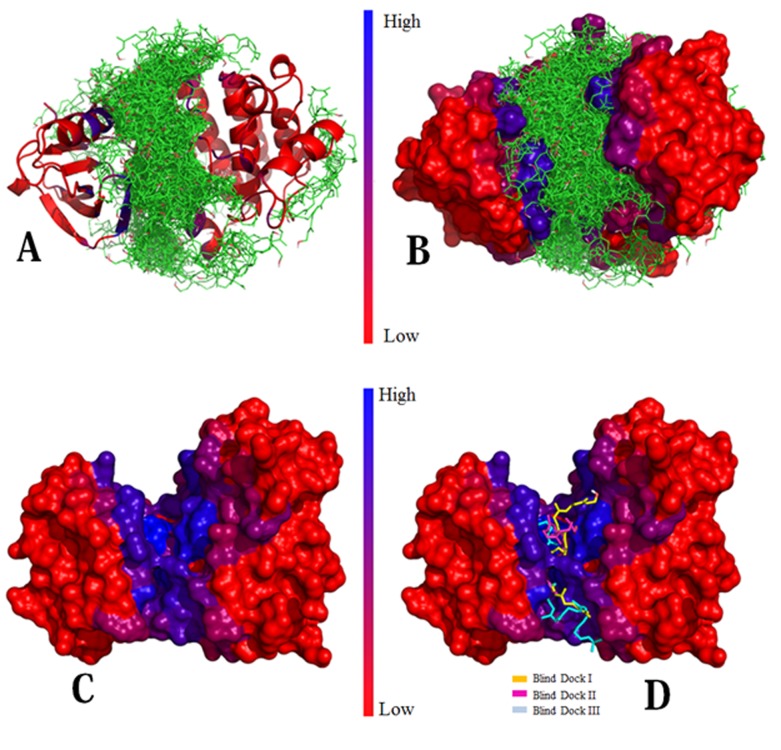
(A, B) FAK structure representation using color based on LBCSR score showing all conformations for docking; (C) FAK structure with color based on LBCSR score; (D) Selected structure (based on binding energy) from the three docking experiments.

**Figure 3:**
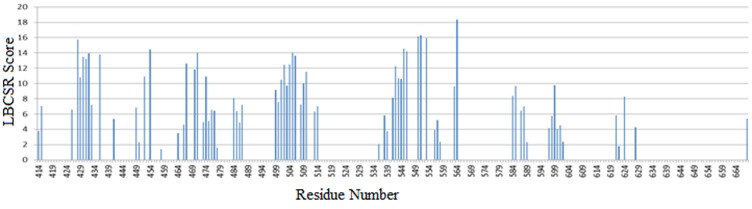
Calculated average LBCSR scores for all the residues of Focal Adhesion Kinase for three experiments.

Based on the LBCSR score calculated for all three experiments, the scores for all the residues were plotted as graph (Figure 3) as well as plotted as false color on the 3D structure of the protein (Figure 2). From the LBCSR score it can be inferred that the ligand shows a high affinity towards the region with Asp564, Asn551, Arg550, Leu553 and Ile428, respectively.

### Refined docking

All the residues with more than LBCSR score was considered for the active site prediction. A total of 77 residues were found to be above and incidentally which also forms the ATP binding site and the catalytic loop (546-551) and formed between the N and C lobe.

The centre of geometry (coordinates = 10.8, 1.0, 15.0) of these 77 residues was considered for the centre of the binding pocket of Solanesol. A grid of size 60 x 62 x 68 was considered to exactly fit all the 77 residues. Autodock4.2 was again used for docking of Solanesol with FAK with this grid setting for two more times, first time with default setting for 200 conformations and later docked with exhaustive setting for the same number of conformations. All 400 conformations were rescored using DSX online server with CSD settings (Table 3).

### Analysis of Final Docked structure

**Figure 4:**
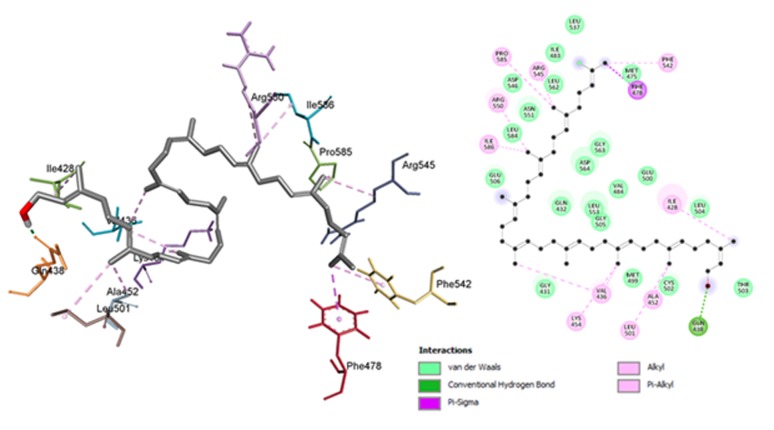
3D (A) and 2D (B) mapof Solanesol when bound to Focal adhesion kinase (FAK) showing different kinds of interactions.

The hydroxyl end of Solanesol binds to the binding site of ATP and interacts with Ile428, Val436, Ala452, Lys454 and Leu501 (Figure 4). These residues that forms the binding pocket for ATP, forms Alkyl-Pi hydrophobic interactions with the double bonds of the ligand. Where the isobutyl end of the ligand gets attached to the Catalytic loop and αC helix. The ligand interacts with Phe542, Arg545, Arg550 (Catalytic loop) and Phe478 (αC helix terminal) with Alkyl and Sigma-Pi interactions. Pro585, Ile586 near the ATP active site also forms similar hydrophobic interactions with the ligand. Oxygen of Solanesol forms a very conventional H-bond with OE1 of Gln438, which is very near to G-Loop and ATP binding site.

These 12 hydrophobic interactions of 11 residues with Solanesol stabilize the ligand at the middle of N and C lobe of FAK. As the ligand share interacting residues with both the side it may be act as a better inhibitor for FAK. This structure was used for molecular dynamics studies.

### Analysis of Molecular Dynamics

**Figure 5:**
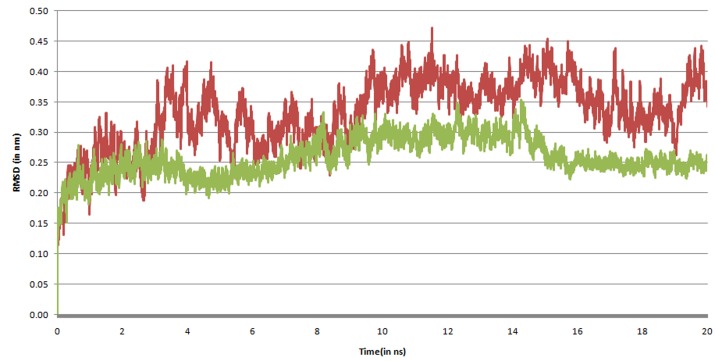
Root mean square deviation (RMSD) of Focal adhesion kinase backbone atoms when bound to Solanesol. It gets stabilized nearly after 16ns of simulation.

For the measuring the stability, the RMS deviation in the backbone of the Solanesol bound FAK and only FAK been measured and plotted in accordance to time (Figure 5). After an initial instability, the backbone changes its shapes linearly with a linear slope increase in RMSD between 4.5ns to 7.7ns after again a short destabilization the system vaguely equilibrates from 9.4ns to 14.2ns. It takes the system almost 15ns to equilibrate, after which the system maintains it position and shape.

**Figure 6:**
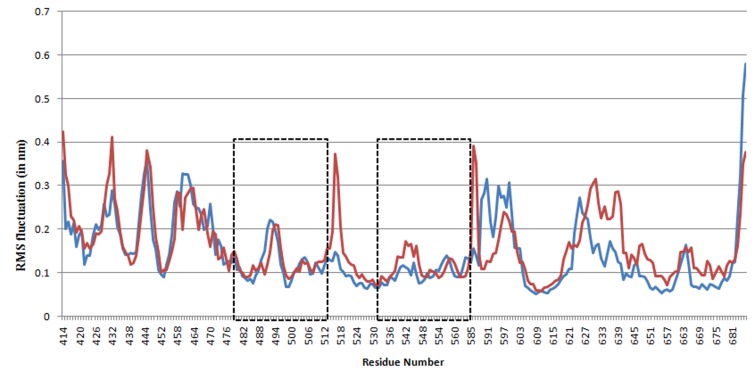
RMS Fluctuation (in nm) in alpha carbon atom of each residue in FAK (Red) and Solanesol bound FAK (Blue). Boxes show the binding site region of FAK.

Comparison of the RMSD of both the trajectories from the minimized structures shows, Solanesol bound FAK backbone show more stability than that of independent FAK backbone. RMSF (Root mean Square Fluctuation) of Solanesol bond FAK exhibits less fluctuation at the places where Solanesol is bond (Figure 6).

Total solvent accessible surface area (SASA) was checked by g_sas tool of Gromacs for analyzing the change in surface area with respect to time. It show a quite negative correlation with the RMSD suggesting the better binding of the ligand leads to a lower surface area of the protein thus closing the active site for any further contact. It remains stable for the wider part of 6ns - 18ns range after which the backbone gets stabilized and may also affect the surface area.

### MM-PBSA Calculation

**Figure 7:**
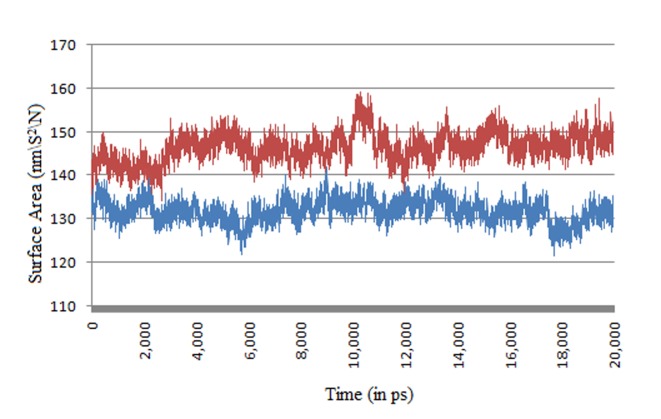
Total Solvent Accessible Surface Area of Focal Adhesion Kinase with (Blue) and without (Red) bound Solanesol.

**Figure 8:**
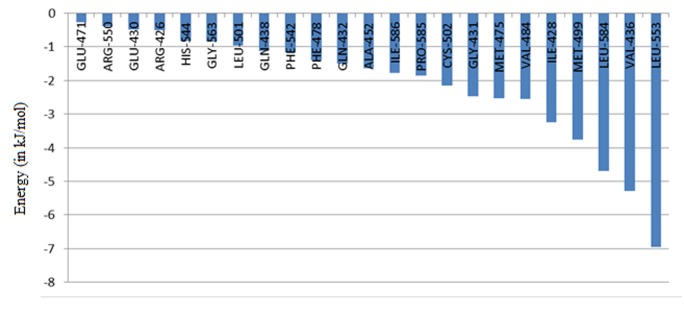
MM-PBSA based residue energy profile for active site residues.

Binding site residues for Solanesol were selected by taking 3.5A radius from Solanesol. Molecular mechanics Poisson-Boltzmann surface area (MM-PBSA) was calculated for the last 5 ns with 20 ps steps for the binding site residues versus Solanesol using g_mmpbsa tool. Per residue analysis of the result was done and plotted using the script provided. This analysis suggests that, Ile428, Gly431, Val436, Val484, Met499, Cys502 and Leu553 interacts most favorably with the ligand (Figure 7). Interestingly these all residues are part of ATP binding pocket of FAK. Gly431 is a part of the G-loop, which helps in the phosphorylation of the protein, also interacts favorably along with Leu584, which is part of the activation loop. A very low ΔΔG, of -113.85 kJ/Mol (Table 4), proves solanesol have a very high binding affinity towards the FAK structure. The change in binding energy with time was also plotted (Figure 8). It shows that the ligand gets stabilized with a very high binding affinity after 17 ns of simulation.

## Conclusion

We report the binding of Solanesol with FAK using a GROMOS96 (53a6) force-field simulated docked model of Solanesol-FAK complex. The binding of solanesol at the ATP binding site to inhibit the phosphorylation of FAK is explained. Data help to understand binding of flexible compounds with FAK for potential inhibition.

## Figures and Tables

**Table 1:** Blind docking analysis with three different experiments (with different starting conformations) using standard autodock protocol

Experiment	Total Conformations	Grid Center	L.B.E (ΔG) in kCal/Mol	Total Cluster	Highest number of member in any cluster	Average B.E of cluster with most number of members
Blind Docking I	500	Center of Protein	-7.08	328	10	0.23
Blind Docking II	200	Center of Protein	-8.54	160	5	-0.19
Blind Docking III	200	Center of Protein	-5.68	187	3	0.13

**Table 2:** Top residues with best LBCSR scores of all three experiments

Residues	Blind Docking I	Blind Docking II	Blind Docking III
LYS-454	5.66	16.5	11.27
ASP-564	8.89	22.35	14.77
ARG-550	7.48	16.33	13.64
GLU-500	2.48	12.06	6.14
ARG-426	3.04	12.11	3.18
GLU-430	5.02	13.87	13.49
LEU-501	4.25	11.98	8.23
GLY-563	4.25	12.08	6.76
GLU-471	6.78	22.5	9.55
GLN-432	5.65	21.14	11.85
ALA-452	4.88	12.3	7.36
CYS-502	5.31	12.32	9.13
PRO-585	4.09	15.5	7.17
HIS-544	4.98	15.97	9.29
GLY-431	5.12	17.82	11.71
VAL-484	3.74	8.93	5.78
MET-475	2.08	10.95	0.69
ILE-428	5.79	16.92	13.77
MET-499	4.42	10.3	5.78
LEU-584	2.3	13.2	10.42
VAL-436	5.55	15.14	11.36
LEU-553	6.3	17.18	13.47

**Table 3:** DSX, knowledge based scoring of Exhaustive and normal Docking

	Total Score	Per Contact Score	Torsional Score	SAS Score	Binding Free Energy
Exhaustive Parameter	-166.61	-0.19	-11.13	-16.1	-7.3
Default Parameter	-131.69	-0.17	-12.43	-8.59	-7.25

**Table 4:** MM-PBSA based final Binding free energy of Solanesol with Focal Adhesion Kinase

ΔG-vdw (in kJ/Mol)	ΔG-electro (in kJ/Mol)	ΔG-polar (in kJ/Mol)	ΔG-SAS (in kJ/Mol)	ΔΔG-BE (in kJ/Mol)
-145.35	-1.71	52.05	-18.85	-113.85

**Figure 1:**
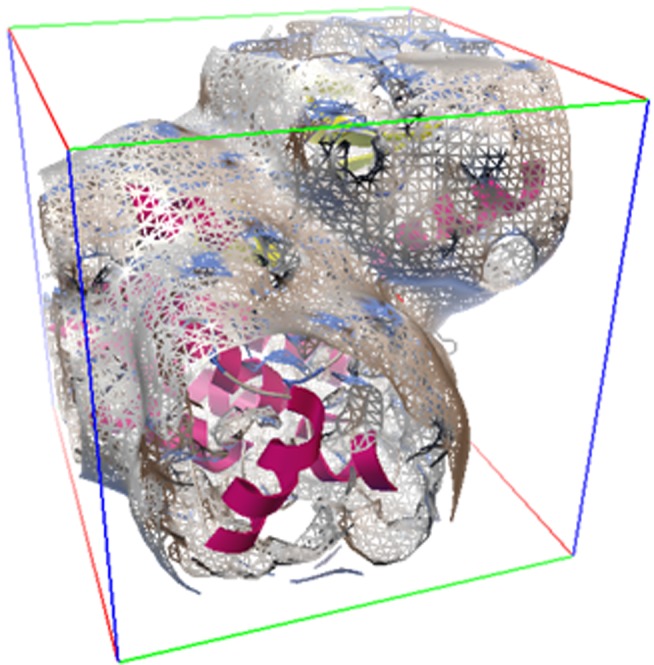
An autoGrid map on FAK generated by AutoGrid 4 for blind docking is shown.

**Figure 9:**
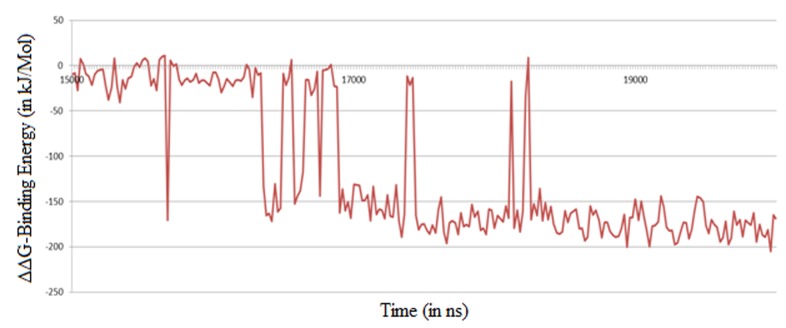
Binding Energy (ΔΔG) [kJ/mol]) using MM-PBSA method with respect to time
